# Inadequacies of musculoskeletal medicine curriculum for undergraduate medical students: a cross-sectional study

**DOI:** 10.1590/1516-3180.2019.0526.R1.19022020

**Published:** 2020-06-22

**Authors:** Delio Eulalio Martins, Ana Cristina Kuhn Pletsch Roncati, Robson Oliveira Rocha, Marcos Paulo Freire

**Affiliations:** I MSc, PhD. Professor and Coordinator, Universidade Anhembi Morumbi, São Paulo (SP), Brazil.; II MSc. Academic Manager, School of Health Sciences, Universidade Anhembi Morumbi, São Paulo (SP), Brazil.; III PhD. Coordinator of Medical Course, Universidade Anhembi Morumbi, São Paulo (SP), Brazil.; IV PhD. Director, School of Health Sciences, Universidade Anhembi Morumbi, São Paulo (SP), Brazil.

**Keywords:** Education, Learning, Curriculum, Teaching methods, Educational models, Orthopedic curriculum, Musculoskeletal curriculum, Medical curriculum, Competency-based curriculum

## Abstract

**BACKGROUND:**

Musculoskeletal disorders account for up to one in four of general-practice consultations and almost one third of complaints in primary-care clinical practice. However, an insufficient amount of time and importance is given to their teaching in most medical schools.

**OBJECTIVE:**

To evaluate the acquisition of musculoskeletal competences in our institution, in order to identify flaws and propose changes to correct and improve the musculoskeletal curriculum.

**DESIGN AND SETTING:**

Cross-sectional study conducted in São Paulo, Brazil.

**METHODS:**

First to fifth-year medical students were enrolled in a survey using the Freedman and Bernstein musculoskeletal examination, in order to evaluate the acquisition of musculoskeletal competencies. Categorical data were analyzed using the chi-square test. Continuous data were analyzed using one-way analysis of variance (ANOVA). The level of significance was set as P < 0.05.

**RESULTS:**

A total of 545 students completed the questionnaire: from year 2, 115/167 (29.6%); from year 3, 118/138 (30.4%); from year 4, 98/130 (25.3%); and from year 5, 57/110 (14.7%). None of the students achieved the pass mark (established as 70%). The level of confidence in performing musculoskeletal examination was very low (3.7 ± 2.2; n = 386) and bore no relationship to the percentage of correct answers in the questionnaire (r = 0.331; 95% confidence interval, CI: 0.239-0.417; P < 0.001).

**CONCLUSION:**

Undergraduate teaching is the only exposure most general practitioners have to orthopedic problems. Universities are concerned about the adequacy of the musculoskeletal programs taught in their institutions. Student scores were found to be unsatisfactory in all the topics evaluated.

## INTRODUCTION

Musculoskeletal disorders account for up one in four general-practice consultations^1^ and almost one third of complaints in primary-care clinical practice. However, an insufficient amount of time and importance is given to their teaching in most medical schools.^2^^,^^3^ Moreover, the knowledge acquired is not always in line with what professors desire or plan. Active techniques have been included in undergraduate training as a powerful teaching tool for improving the quality of learning.

Knowledge of the basis of musculoskeletal disorders is fundamental for general practitioners, family practitioners, pediatricians, emergency physicians, interns and, of course, rheumatologists and orthopedists. Thus, a very well-structured curriculum is necessary in order to achieve the competences desired.

One way to evaluate the basic competency attained by medical school students in relation to the musculoskeletal system is the Freedman and Bernstein examination. This was developed and validated by 124 chairs of orthopedic residency programs in the United States and the pass mark for physicians has been set at 70%.^4^

## OBJECTIVE

The objective of this study was to evaluate the acquisition of musculoskeletal competences in our institution, in order to identify flaws and propose changes to correct and improve the musculoskeletal curriculum.

## METHODS

Aspects of the musculoskeletal system are taught a little at a time each year up to the end of the fourth year in our medical school. Thus, second to fifth-year medical school students were enrolled in a survey in which they were asked to complete the Freedman and Bernstein musculoskeletal examination^4^ and to fill in a form containing questions regarding demographic information, including their year of training, personal preferences among subspecialties in medicine (clinical area of interest) and feelings about the time spent on theoretical and practical classes during the whole period of musculoskeletal training that they had had up to that moment.

To assess the students’ perceptions regarding their classes, a five-point bipolar measurement scale (five categories) centered on “indifferent” was used. The five categories were: far too many classes (the number could be reduced); good number of classes (not too many and not too few); reasonable number of classes (enough, but more classes would be welcome); insufficient number of classes (more classes definitely needed); very poor number of classes (not enough time dedicated to classes)

The types of active teaching methodologies that the students had had over the course of their undergraduate studies up to that point, and the percentage of each type, were assessed.

A tool asking about their confidence in performing orthopedic physical examinations and making diagnostic hypotheses for musculoskeletal disorders was applied using a 10-point scale. The confidence scores was grouped as 0-3 (low), 4-7 (moderate) and 8-10 (high).

The testing was performed with the cooperation of the professors of each year. Written informed consent was obtained from the participants and the examination was anonymous. No time limit was imposed.

The distribution of academic content, according to the semester taught, is shown in Table 1. Anatomy content is taught by the end of the second year, while major clinical and therapeutic content is taught by the end of the fourth year.

**Table 1.** Contents of the Freedman and Bernstein musculoskeletal questionnaire according to the semester taught and curricular unit

Question	Semester (curricular unit)	1	2	3	4	5	6	7	8
	Topic								
1	Congenital dislocation of the hip					MPV		OT	
2	Compartment syndrome	Mo		LS			GS	OT	
3	Arthritis (septic or inflammatory)				MPIV			OT	
4	Knee displacement			LS				OT	
5	Open fracture							OT	
6	Low back pain (differential diagnosis: tumor/infection)				MPIV			OT	
7	Compartment syndrome	Mo					GS	OT	
8	Upper limb fractures (scaphoid)			LS				OT	
9	Hip dislocation							OT	
10	Carpal tunnel syndrome (clinical and anatomical)			LS	MPIV			OT	Ne
11	Disc herniation; orthopedic and neurological propaedeutics				MPIV			OT	
12	Anatomy of peripheral nerves				NS			OT	
13	Fractures and ligament sprain in children							OT	
14	Low back pain				MPIV		AH	Rh	
15	Anatomy of peripheral nerves in lower limbs				NS				
16	Knee effusion and hemarthrosis							OT	
17	Bone tumor							OT	
18	Rheumatoid arthritis and osteoarthrosis							Rh	
19	Bone tumor or myeloma							OT	
20	Anatomy of knee ligaments			LS				OT	
21	Osteoporosis and/or osteomalacia				MPIV	MPV	AH	Rh	
22	Proximal femur fracture and/or hip vascular anatomy			LS				OT	
23	Anatomy of upper limb muscles and/or lateral epicondylitis			LS				OT	
24	Anatomy of upper limb muscles			LS					
25	Anatomy of upper limb muscles and/or rotator cuff			LS				OT	

Mo = morphology; LS = locomotor system; NS = nervous system; MPIV = medical practices IV; MPV = medical practices V; GS = general surgery and anesthesiology; AH = adult health; OT = orthopedics and traumatology; Rh = rheumatology; Ne = neurology.

The general characteristics of the sample are shown in Table 2. In total, 388 (71.2%) out of 545 students completed the questionnaires. The split according to year was as follows: year 2 = 115/167 (29.6%); year 3 = 118/138 (30.4%); year 4 = 98/130 (25.3%); and year 5 = 57/110 (14.7%).

**Table 2.** General characteristics of the sample

	Year 2	Year 3	Year 4	Year 5
Number of students	115	118	98	57
Gender				
Male	27	32	35	19
Female	88	86	63	38
Mean age (years)	21.3	22.8	23.6	25.1

The Freedman and Bernstein examination was developed and validated to test how well medical school graduates understood basic musculoskeletal problems.^4^ The questionnaire consists of 25 short open questions about important topics such as fractures, tumors, dislocations, back pain, arthritis and emergencies that need to be recognized by general physicians so that patients with these conditions can be referred to an orthopedic surgeon immediately.

The examination was scored anonymously using an answer key. The pass mark was set as 70%, based on recommendations from previous studies.^4^^,^^5^ Each question was worth a maximum of one point and the raw scores were multiplied by four to obtain a final score between zero and 100.

The lesson plans of the previous year were evaluated and used as a reference to determine whether the topic had been taught to the students and in which year of the medical curriculum this had been done.

The results were analyzed using the R software (version 3.3.2, 2016; Vienna, Austria) and graphs were compiled using the ggplot2 package. Descriptive data and confidence intervals were determined. Categorical data were analyzed using the chi-square test. Continuous data were analyzed using one-way analysis of variance (ANOVA). The significance level was set as P < 0.05.

## RESULTS

**Figure 1.**
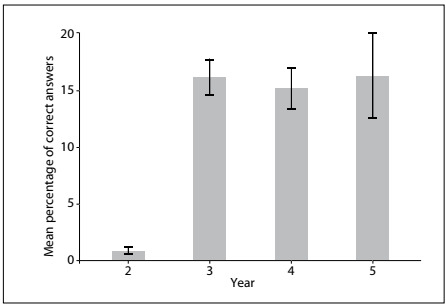
Distribution of correct answers for each school year. Results expressed as mean percentage and 95% confidence interval.

None of the students achieved the pass mark, which had been established as 70%. There was no difference in the percentage of correct answers between the third-year students (16.2 ± 9) and the fifth-year students (16.3 ± 14.4). The students’ overall performance was very low (Figure 1).

Out of all the questions, the first question (What common problem must all newborns be examined for?) received the most correct responses (49.7% ± 50.1), while question 11 (A patient had a disc herniation pressing on the fifth lumbar nerve root. How is motor function of the fifth lumbar nerve root tested?) received the fewest correct responses (0.5% ± 7.7).

**Figure 2.**
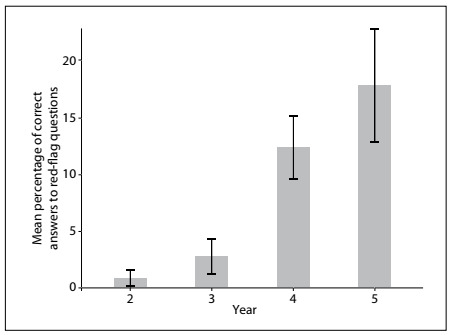
Distribution of correct answers to red-flag questions according to school year. Data expressed as mean and 95% confidence interval.

**Figure 3.**
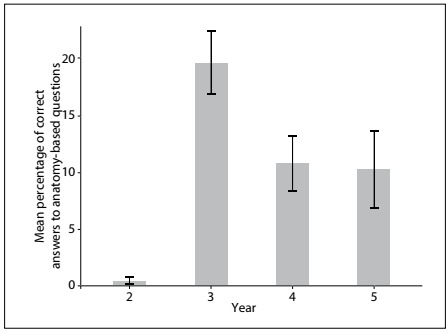
Distribution of correct answers to basic anatomy questions according to school year. Data expressed as mean and 95% confidence interval.

Based on the hypothesis that students starting out in medical school would perform better in relation to questions of basic anatomy while students in the later years would score better in relation to important clinical questions, a group component score was obtained by forming the following groups: anatomy-based questions (numbers 8, 10, 11, 12, 15, 20, 22, 23, 24 and 25); “red-flag” questions (numbers 2, 4, 5, 6 and 7); and miscellaneous questions (numbers 1, 3, 9, 13, 14, 16, 17,18, 19 and 21). Red-flag questions related to situations that are considered to be clinical emergencies, in which non-recognition can cause irreparable harm to the patient. They were answered most successfully by the fifth-year students (Figure 2), albeit with a low incidence of correct answers. On the other hand, basic anatomy questions were answered most successfully by the third-year students, and the percentage of correct answers decreased over the subsequent years (Figure 3).

**Figure 4.**
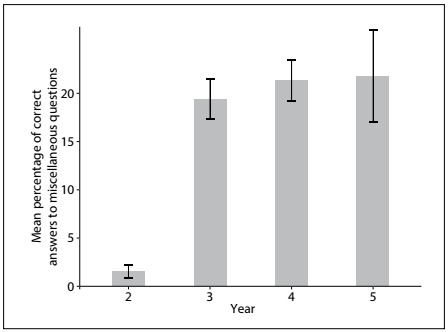
Distribution of correct answers to miscellaneous questions according to school year. Data expressed as mean and 95% confidence interval.

Excluding second-year students, no difference in the proportion of correct answers was found in relation to the miscellaneous questions among the other school years (Figure 4).

The level of confidence in performing musculoskeletal examination was very low (3.7 ± 2.2; n = 386) and bore no relationship to the percentage of correct answers in the questionnaire (r = 0.331; 95% confidence interval, CI: 0.239-0.417; P < 0.001). The level of confidence in performing physical examination was highest in the third year (4.8 ± 1.7; n = 118).

The students’ perceptions of the teaching methods used by professors and the amounts of time spent on theoretical and practical classes are shown in Table 3. The majority (83.7%) of the students considered that the amount of time spent on theoretical classes was reasonable or good (83.7%). Theoretical classes were the most commonly used teaching methodology (44.5% ± 23.4; n = 349).

**Table 3.** Students’ perceptions of the types of class per school year

Class mode		Year					
		2 (n = 115)	3 (n = 118)	4 (n = 98)	5 (n = 57)	Total (n = 388)	P-value
Theoretical class time	Very poor number of classes	4 (3.5%)	8 (6.8%)	13 (13.3%)	14 (25.5%)	39 (10.1%)	< 0.001
	Insufficient number of classes	4 (3.5%)	1 (0.8%)	4 (4.1%)	6 (10.9%)	15 (3.9%)	
	Reasonable number of classes	59 (51.3%)	52 (44.1%)	49 (50%)	19 (34.5%)	179 (46.4%)	
	Good number of classes	46 (40%)	56 (47.5%)	30 (30.6%)	12 (21.8%)	144 (37.3%)	
	Far too many classes	2 (1.7%)	1 (0.8%)	2 (2%)	4 (7.3%)	9 (2.3%)	
Practical class time	Very poor number of classes	18 (15.8%)	12 (10.2%)	25 (25.5%)	15 (27.3%)	70 (18.2%)	< 0.001
	Insufficient number of classes	7 (6.1%)	4 (3.4%)	12 (12.2%)	17 (30.9%)	40 (10.4%)	
	Reasonable number of classes	52 (45.6%)	48 (40.7%)	43 (43.9%)	16 (29.1%)	159 (41.3%)	
	Good number of classes	37 (32.5%)	52 (44.1%)	13 (13.3%)	6 (10.9%)	108 (28.1%)	
	Far too many classes	0 (0%)	2 (1.7%)	5 (5.1%)	1 (1.8%)	8 (2.1%)	
TBL (mean ± SD)		20.2 ± 25.9 (n = 104)	13.5 ± 12.3 (n = 115)	9.4 ± 12.5 (n = 91)	17.9 ± 14 (n = 40)	14.9 ± 18.1 (n = 350)	< 0.001
PBL (mean ± SD)		10.7 ± 19.6 (n = 103)	6 ± 9.8 (n = 115)	5.8 ± 14.3 (n = 91)	14.2 ± 17.6 (n = 40)	8.3 ± 15.5 (n = 349)	0.004
Case study (mean ± SD)		21.6 ± 23.2 (n = 104)	11.9 ± 12.4 (n = 114)	9.1 ± 12.3 (n = 90)	11 ± 13 (n = 40)	14 ± 17.2 (n = 348)	< 0.001
Dialogued lecture class (mean ± SD)		48.1 ± 24.9 (n = 104)	38.9 ± 21.4 (n = 115)	40.4 ± 20.5 (n = 91)	61.5 ± 22 (n = 39)	44.5 ± 23.4 (n = 349)	< 0.001
Laboratory classes (mean ± SD)		36 ± 27.2 (n = 104)	26.9 ± 18.5 (n = 115)	24.2 ± 18.7 (n = 91)	17.9 ± 18.7 (n = 39)	27.9 ± 22.2 (n = 349)	< 0.001
Practice with patients (mean ± SD)		17.3 ± 24.6 (n = 104)	16.2 ± 15.2 (n = 115)	16.6 ± 16.8 (n = 90)	15.2 ± 13.1 (n = 39)	16.5 ± 18.6 (n = 348)	0.935
Body painting (mean ± SD)		9.3 ± 13.2 (n = 104)	5.2 ± 6.4 (n = 114)	7.5 ± 11.8 (n = 90)	8.4 ± 10.7 (n = 39)	7.4 ± 10.8 (n = 347)	0.038
Peer-to-peer (mean ± SD)		10 ± 17.6 (n = 103)	6.1 ± 9.8 (n = 115)	5.9 ± 7.4 (n = 91)	8.4 ± 12.8 (n = 39)	7,5 ± 12.6 (n = 348)	0.066
Others (mean ± SD)		0.1 ± 0.7 (n = 104)	0.1 ± 1.1 (n = 115)	1.2 ± 4.3 (n = 91)	0.5 ± 2.2 (n = 39)	0.4 ± 2.5 (n = 349)	0.008

TBL = team-based learning; PBL = problem-based learning; SD = standard deviation.

## DISCUSSION

In this study, we evaluated students in the second to the fifth academic years of medical school using a survey based on the Freedman and Bernstein questionnaire, to analyze their progress in achieving musculoskeletal competencies. None of the students attained the pass mark of 70%. Fifth-year students performed better on red flag questions while third-year students performed better on anatomy questions. The students’ level of confidence in performing musculoskeletal examinations was very low (< 5, on a scale of 0-10 points).

The burden of musculoskeletal problems within primary-care medical practice and on healthcare resources is well known.^6^^,^^7^^,^^8^ However, undergraduate teaching is the only exposure that the majority of general practitioners will have to orthopedic problems. Many universities are concerned about the adequacy of the musculoskeletal programs taught in their institutions.^1^^,^^4^^,^^5^^,^^7^^,^^8^^,^^9^^,^^10^^,^^11^ The present study serves to aid in understanding and proposing changes since our students correctly answered fewer than 20% of the questions.

However, it is important to look not only at the curriculum but also, and sometimes even more importantly, at the way in which the curricular content is being taught. At our institution, we use the spiral curricular model, in which students see content more than once (Table 2). However, although active methodologies are used, students are not retaining that knowledge.

Third-year students performed better on basic or anatomical questions, which they had just finished studying through the spiral curriculum, but the level of correct responses decreased over the subsequent years. This may have been due to many factors, such as the methodologies used or differences in the way in which the content was taught, since some changes to the teaching staff occurred during this period.

Fifth-year students performed better in the so-called red-flag set of questions. This was because the major clinical and therapeutic content had been taught that year. Unfortunately, students in the sixth year were not evaluated in this study: this would have enabled analysis on the students’ learning.

Attention needs to be given to curricular competencies. In Brazil, competencies have been well described in relation to the medical curriculum but not for curricular subjects.^12^ Thus, there is no standardization regarding the musculoskeletal curriculum for all universities and each professor or institution can decide what is important to teach, and sometimes they do not cover all the core subjects. There is also the possibility that professors are not fulfilling the lesson plan. Since this study was conducted in only one institution, we are unable to say whether this is the case throughout the country, but our study sheds light on an area that deserves attention.

## CONCLUSION

In summary, the way in which musculoskeletal disorders are being taught in medical schools today needs to be reviewed. There is scope for progress in relation to some points, such as the standardization of content, commitment of teachers to teaching this content, improvement of active teaching methodologies, use of sound in-depth lesson plans and supervision and confirmation that these plans are being fulfilled.
